# Optical coherence tomography evaluation of choroidal structure changes in diabetic retinopathy patients: A systematic review and meta-analysis

**DOI:** 10.3389/fmed.2022.986209

**Published:** 2022-10-20

**Authors:** Jikuan Jiang, Jingyuan Liu, Jia Yang, Bing Jiang

**Affiliations:** ^1^Department of Ophthalmology of Second Xiangya Hospital, Central South University, Changsha, China; ^2^Hunan Clinical Research Center of Ophthalmic Disease, Changsha, China

**Keywords:** diabetic retinopathy, choroidal vascularity index, choroidal thickness, choroidal structures, optical coherence tomography, meta-analysis

## Abstract

**Introduction:**

Diabetic retinopathy (DR) is one of the major causes of blindness among working-aged adults worldwide. This study aimed to evaluate the differences in the subfoveal choroidal thickness (SFCT) and choroidal vascularity index (CVI) using optical coherence tomography (OCT) of patients with diabetic eyes with no retinopathy (NDR) and with diabetic retinopathy (DR).

**Methods:**

We performed a comprehensive literature search of the PubMed, Embase, and Cochrane Library databases up to October 2021. The weighted mean difference (WMD) with the 95% confidence interval (CI) was pooled for continuous outcomes.

**Results:**

Twenty-three cross-sectional studies comprising 2,534 eyes including 1,070 NDR eyes, 1,464 DR eyes were included in the systematic review and meta-analysis. The pooled results showed SFCT was significantly thicker in DR than in NDR patients after adjusting for axial length (WMD = 27.90 μm; 95% CI: 11.51 to 44.28; *P* = 0.001), and the CVI was significantly lower in DR patients (WMD = −1.59; 95% CI: −2.67 to −0.52; *P* = 0.004).

**Conclusion:**

We described changes in the SFCT and CVI in DR. Resultantly, the CVI and SFCT may be valuable parameters for monitoring the onset of DR and helpful for a better understanding of the role of the choroid in the pathological process of DR.

**Systematic review registration:**

https://www.crd.york.ac.uk/prospero/#myprospero, CRD42021228738.

## Introduction

Diabetic retinopathy (DR) is a major cause of blindness in working-aged adults worldwide (1). Since more than one-third of diabetes patients will develop DR in their lifetime, it is important to identify DR in its early stages (2). The pathogenesis of DR involves long-term exposure to hyperglycemia and other systemic risk factors, such as hypertension, ultimately leading to microvascular damage (e.g., disruption of the blood-retina barrier) and retinal dysfunction (development of neovascularization) (3). Changes in the retinal vascular structure and function, such as widening of the retinal arterioles and microvascular dysfunction, are closely related to DR, and may lead to capillary wall dilatation (microaneurysms), leakage (edema and hard exudates), and rupture (hemorrhage) (3). The choroid plays a crucial role in the pathophysiology of various retinal diseases, including DR. Since the choroid is a highly vascularized tissue that provides most of the blood supply to the outer retina, including the retinal pigment epithelium (RPE) cells and photoreceptors (4), changes in choroidal structure may play a significant role in the onset of DR (5). In regions with no retinal vasculature, such as the macular, blood supply from the choroid seems far more important. Thus, dysfunction of the choriocapillaris may induce severe harm to the retinal tissue, especially the macula fovea (6).

Until recently, the choroid could only be evaluated using indocyanine green angiography, laser Doppler flowmetry, and ultrasonography, all of which are either invasive or unable to acquire a high-resolution image (2). Optical coherence tomography (OCT) is a non-invasive technique that allows ophthalmologists to evaluate morphological features of the retina. Recently, spectral-domain optical coherence tomography (SD-OCT), with enhanced depth imaging (EDI) software and swept source OCT (SS-OCT), has been commonly used as a non-invasive imaging modality, which is able to acquire high-resolution images to quantitively assess the choroidal structure (7).

Nonetheless, it is unknown what changes occur in the choroid in eyes of DM (diabetic mellitus) and DR patients. Many studies have used subfoveal choroidal thickness (SFCT) and choroidal vascularity index (CVI) as quantitative metrics. DR eyes reportedly have a decreased CVI compared with that in eyes with no diabetic retinopathy (NDR) (5, 8, 9).

However, when comparing SFCT between diabetes patients with DR and NDR, studies have reported diverged findings. Several studies have reported a thinning of the choroid in DR eyes compared to that in NDR (10), while others reported a thickening (11–13) or no change (14–16).

Therefore, a meta-analysis can help provide reliable data to elucidate these findings.

To better understand the role of choroid in the pathological process of DR, we perform a meta-analysis to systematically evaluate the measurements of SFCT and CVI using OCT in diabetic eyes with NDR and DR and determine whether change of SFCT and CVI can correlated with the onset of DR in diabetes mellitus patients.

## Methods

### Literature search

To identify studies of relevant, we conducted a systematic review of the literature. We searched the PubMed, Embase, and Cochrane Library databases from inception to October 2021. Only articles written in English were included. We used the following medical subject as search items: “subfoveal choroidal thickness,” “SFCT,” “choroidal thickness,” “choroidal vascularity index,” “CVI,” “luminal area,” “stromal area,” “diabetic retinopathy,” “diabetic choroidopathy,” “optical coherence tomography,” and “OCT.” The last search was conducted in October 2021. Moreover, the reviewers manually reviewed the reference lists of relevant published articles for any additional relevant studies.

### Inclusion/exclusion criteria

The inclusion criteria were: (1) studies enrolling DM patients with NDR and DR; (2) studies using OCT; (3) studies evaluating the subfoveal choroidal thickness or choroidal vascularity index in DM patients with DR and DM without DR; (4) studies reporting the SFCT or CVI with mean and standard deviations, or if it was possible, to measure them from the data presented in the studies by our own calculation.

The exclusion criteria were: (1) conferences, case reports, comments, or reviews; (2) inclusion of subjects who received focal treatment for DR in the last 3 months such as focal laser photocoagulation, panretinal photocoagulation, intravitreal anti-vascular endothelial growth factor or steroid injections, eye diseases that could affect retinal or choroidal anatomy, age <18 years, pregnancy, and high myopia. (3) unavailability of the outcome values for meta-analysis; (4) inability to obtain relevant data for meta-analysis even after contacting the articles' authors; and (5) duplicated data. The screening process was performed separately by two reviewers, and disagreements were resolved by discussion.

### Data extraction

Two reviewers extracted the required information from eligible studies. The extracted data included the following: (1) first author; (2) publication year; (3) study design; (4) origin of study; (5) type of OCT instrument; (6) sample size; (7) age; (8) axial length; (9) DM type; (10) stage of DR; (11) SFCT and CVI assessment protocol; (12) Mean and standard deviation of the SFCT and CVI of every stage of DR. Disagreements were resolved by discussion between two review authors; if no agreement could be reached, a third author would decide. When necessary, we contacted the authors for further information.

### Quality assessment

The quality of cross-sectional studies was analyzed utilizing the 11-item checklist from the Agency for Healthcare Research and Quality (AHRQ) (17). Two review authors subjectively scored each included study, and any differences were resolved by discussion.

### Statistical analysis

Statistical analysis was performed using Review Manager V5.4.1 (Cochrane Collaboration, London, United Kingdom) and Stata software (version 15.1; StataCorp, College Station, Texas). A value of *p* < 0.05, was considered significant, except where otherwise specified. We employed the weighted mean difference (WMD) with a 95% confidence interval (CI) to pool the mean differences in OCT parameters between the NDR and DR groups. The *I*^2^ statistic was used to assess the heterogeneity among studies; values of 25, 25–49% and over 50% were considered low heterogeneity, moderate heterogeneity, and high heterogeneity, respectively (18). A fixed-effects model was used when *I*^2^ <50%; otherwise, a random-effects model was used. Potential publication bias was assessed using funnel plots, Begg's test and Egger test.

## Results

### Search and selection of studies

**Figure 1:**
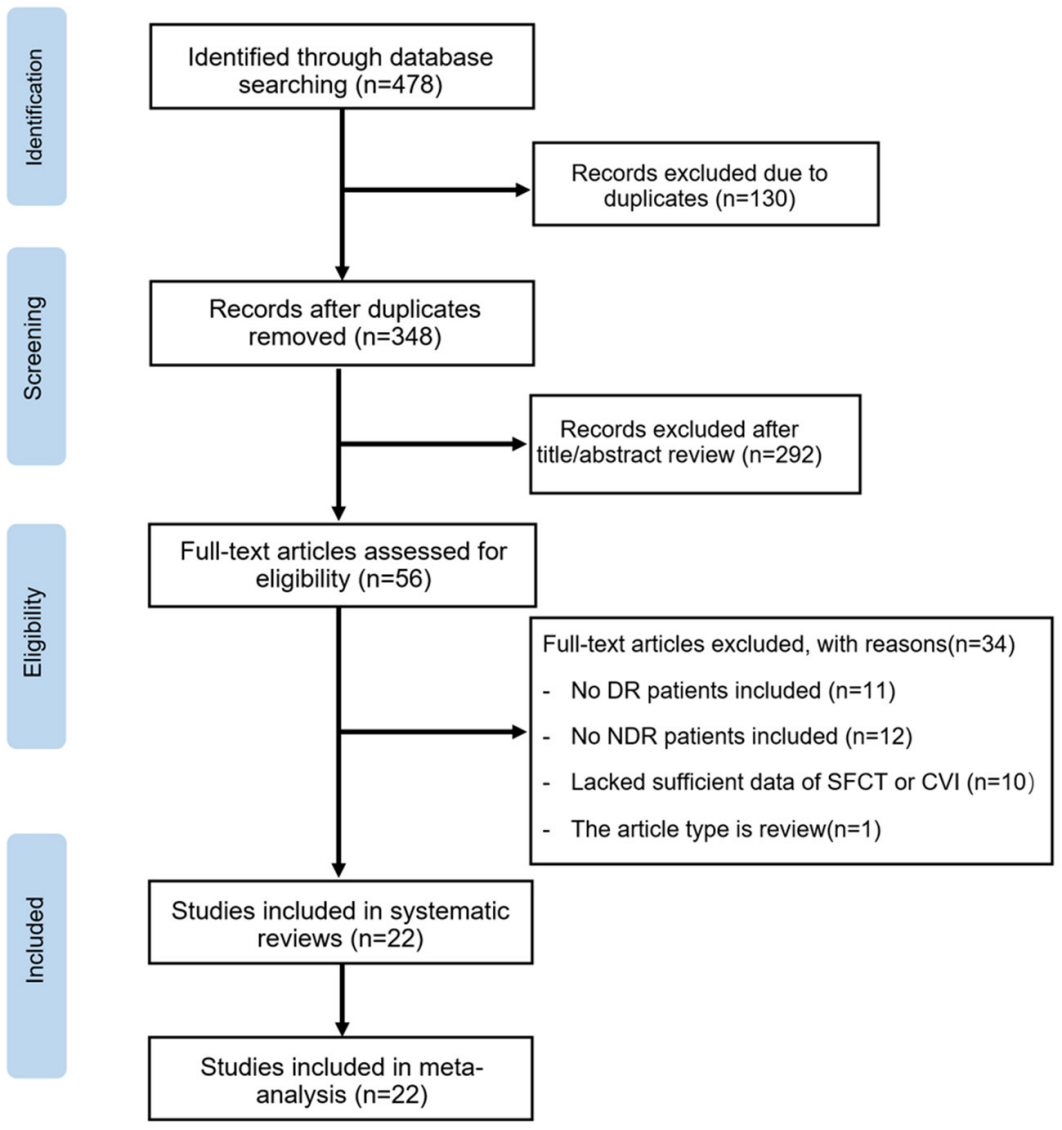
PRISMA flow diagram of the literature search process.

The initial search yielded 478 potentially relevant studies. Of these, 130 articles were excluded due to duplication. Based on titles and abstracts, 292 articles were excluded because of apparently irrelevant. In total, 56 full-text articles were further assessed for eligibility. Of these, 11 articles were excluded based on: no DR patients included in the study, and 12 were excluded for not including NDR patients in the study. Ten articles lacked sufficient data of SFCT and CVI values, and one article was a review. Ultimately, 22 articles met the inclusion criteria and were included in this meta-analysis. The steps of the study selection process and reasons for exclusion are detailed in Figure 1.

### Characteristics of included studies

According to our eligibility criteria, twenty-two cross-sectional studies comprising 2,534 eyes including 1,070 NDR eyes and 1,464 DR eyes, were included in the meta-analysis. Of the twenty-two studies, thirteen were conducted in Asia, eight in Europe, and one in South America. The mean age ranged from 37.0 to 67.9 years. All studies enrolled age-matched subjects with NDR and DR. Eighteen studies employed the SD-OCT, four used SS-OCT. Thirteen studies enrolled m-mNPDR, sNPDR, and PDR subjects, three studies enrolled m-mNPDR and sNPDR subjects, one study enrolled m-mNPDR and PDR subjects, one study only enrolled m-mNPDR subjects, four studies are unavailable for DR stage. The detailed characteristics of the included studies are summarized in Table 1. The AHRQ checklist scores most of the included cross-sectional studies were not <5, demonstrating that the studies were of good quality. The details are presented in Table 2.

**Table 1:** Characteristics of included studies.

**Study**	**Region**	**No. eyes**		**Mean age** ±**SD (yrs)**		**DR stages included**	**OCT**	**Diabetes mellitus types**	**Intraocular treatment history**	**Main outcomes**
		**NDR**	**DR**	**NDR**	**DR**					
Abadia et al. (15)	Spain	49	108	66.2 ± 8.9	67.9 ± 7.6	m-mNPDR, sNPDR, PDR	SS-OCT	Type II	None	SFCT
Abalem et al. (19)	Brazil	42	152	55.4 ± 16.5	60.3 ± 12.3	m-mNPDR, sNPDR, PDR	SD-OCT	Mixed	>3 months	SFCT
Carbonell et al. (20)	Spain	139	103	42.8 ± 10.1	46.8 ± 11.1	m-mNPDR, PDR	SD-OCT	Type I	>6 months	SFCT
Damian et al. (21)	Romania	38	23	57.1 ± 14.4	57.8 ± 11.9	m-mNPDR	SD-OCT	Mixed	None	CVI, SFCT
Endo et al. (11)	Japan	24	68	54.8 ± 14.0	59.5 ± 12.7	m-mNPDR, sNPDR, PDR	SD-OCT	Mixed	None	SFCT
Esmaeelpour et al. (16)	UK	15	36	69.0 ± 10.0	62.5 ± 13.5	m-mNPDR, sNPDR	SD-OCT	Type II	None	SFCT
Esmaeelpour et al. (22)	Austria	15	18	37.0 ± 10.0	39.0 ± 9.0	-	SD-OCT	Type I	None	SFCT
Gupta et al. (23)	India	86	82	-	-	m-mNPDR, sNPDR, PDR	SD-OCT	Mixed	>6 months	CVI
Gupta et al. (24)	India	100	89	61.8 ± 7.5	62.5 ± 6.0	-	SD-OCT	Mixed	>6 months	CVI, SFCT
Horvath et al. (10)	Hungary	17	34	-	-	m-mNPDR, sNPDR, PDR	SS-OCT	Mixed	None	SFCT
Kase et al. (25)	Japan	31	97	57.9 ± 12.9	57.9 ± 11.9	m-mNPDR, sNPDR, PDR	SD-OCT	Mixed	None	CVI
Kase et al. (26)	Japan	18	32	-	-	m-mNPDR, sNPDR, PDR	SD-OCT	Mixed	None	SFCT
Kim et al. (26)	Korea	40	155	62.0 ± 12.4	59.6 ± 12.1	m-mNPDR, sNPDR, PDR	SD-OCT	Mixed	None	SFCT
Kim et al. (12)	Korea	30	89	57.5 ± 15.6	59.4 ± 12.3	m-mNPDR, sNPDR, PDR	SS-OCT	Type II	None	CVI, SFCT
Kinoshita et al. (27)	Japan	40	120	56.9 ± 12.8	59.0 ± 13.0	m-mNPDR, sNPDR, PDR	SD-OCT	Mixed	>6 months	SFCT
Ohara et al. (28)	Japan	14	45	50.7 ± 7.1	50.6 ± 8.1	m-mNPDR, sNPDR, PDR	SS-OCT	Mixed	None	SFCT
Querques et al. (29)	Italy	21	21	65.0 ± 9.0	64.2 ± 10.3	m-mNPDR, sNPDR,	SD-OCT	Type II	None	SFCT
Shen et al. (6)	China	49	34	68.0 ± 6.9	67.8 ± 6.4	m-mNPDR, sNPDR,	SD-OCT	Type II	None	SFCT
Tan et al. (8)	Singapore	25	13	-	-	-	SD-OCT	Mixed	> 3months	CVI
Vujosevic et al. (30)	Italy	22	80	57.6 ± 13.5	57.5 ± 10.8	m-mNPDR, sNPDR, PDR	SD-OCT	Mixed	None	SFCT
Wang et al. (5)	China	22	42	61.8 ± 7.6	62.6 ± 7.6	m-mNPDR, sNPDR, PDR	SD-OCT	Mixed	>2years	CVI
Xu et al. (14)	China	233	23	-	-	-	SD-OCT	Mixed	None	SFCT

NDR, diabetic eyes with no retinopathy; DR, diabetic retinopathy; OCT, Optical coherence tomography; m-mNPDR, mild to moderate non-proliferative diabetic retinopathy; sNPDR, severe non-proliferative diabetic retinopathy; PDR, proliferative diabetic retinopathy; SFCT, subfoveal choroidal thickness; CVI, choroidal vascularity index.

**Table 2:** Methodological quality of included studies.

**Study**	**11-item check list recommended by AHRQ**												
	**i**	**ii**	**iii**	**iv**	**v**	**vi**	**vii**	**viii**	**ix**	**x**	**xi**	**Score**	**Quality**
Abadia et al. (15)	⋆	⋆	⋆	⋆			⋆	⋆				6	M
Abalem et al. (19)	⋆	⋆			⋆	⋆	⋆	⋆				6	M
Carbonell et al. (20)	⋆	⋆	⋆					⋆				4	M
Damian et al. (21)	⋆	⋆	⋆		⋆	⋆		⋆				6	M
Endo et al. (11)	⋆	⋆	⋆		⋆	⋆		⋆				6	M
Esmaeelpour et al. (16)	⋆	⋆			⋆	⋆	⋆	⋆				6	M
Esmaeelpour et al. (22)	⋆	⋆			⋆	⋆	⋆	⋆				6	M
Gupta et al. (23)	⋆	⋆	⋆	⋆	⋆		⋆	⋆				7	M
Gupta et al. (24)	⋆	⋆	⋆	⋆	⋆		⋆	⋆				7	M
Horvath et al. (10)	⋆	⋆	⋆	⋆	⋆			⋆				6	M
Kase et al. (25)	⋆	⋆	⋆	⋆	⋆	⋆		⋆				7	M
Kase et al. (26)	⋆	⋆	⋆		⋆	⋆		⋆				6	M
Kim et al. (13)	⋆	⋆	⋆	⋆	⋆			⋆				6	M
Kim et al. (9)	⋆	⋆	⋆	⋆	⋆	⋆	⋆	⋆				8	H
Kinoshita et al. (27)	⋆	⋆	⋆		⋆	⋆		⋆				6	M
Ohara et al. (28)	⋆	⋆	⋆		⋆	⋆						5	M
Querques et al. (29)	⋆	⋆	⋆	⋆	⋆	⋆		⋆				7	M
Shen et al. (6)	⋆	⋆	⋆		⋆	⋆		⋆				6	M
Tan et al. (8)	⋆	⋆			⋆	⋆	⋆	⋆				6	M
Vujosevic et al. (30)	⋆	⋆	⋆	⋆	⋆	⋆		⋆				7	M
Wang et al. (5)	⋆	⋆			⋆	⋆		⋆				5	M
Xu et al. (14)	⋆	⋆	⋆	⋆	⋆	⋆	⋆	⋆				8	H

AHRQ, Agency for Healthcare Research and Quality; H, high quality; M, moderate quality; L, low quality; high quality (score: 8–11); moderate quality (score: 4–7); low quality (score: 0-3). i, Define the source of information; ii, List inclusion and exclusion criteria for exposed and unexposed subjects (cases and controls) or refer to previous publications; iii, Indicate time period used for identifying patients; iv, Indicate whether or not subjects were consecutive if not population-based; v, Indicate if evaluators of subjective components of study were masked to other aspects of the status of the participants; vi, Describe any assessments undertaken for quality assurance purposes; vii, Explain any patient exclusions from analysis; viii, Describe how confounding was assessed and/or controlled; ix, If applicable, explain how missing data were handled in the analysis; x, Summarize patient response rates and completeness of data collection; xi, Clarify what follow-up, if any, was expected and the percentage of patients for which incomplete data or follow-up was obtained.

The *symbol indicates the score of 11-item AHRQ check list.

### Comparison of the SFCT or CVI between NDR and DR patients

**Figure 2:**
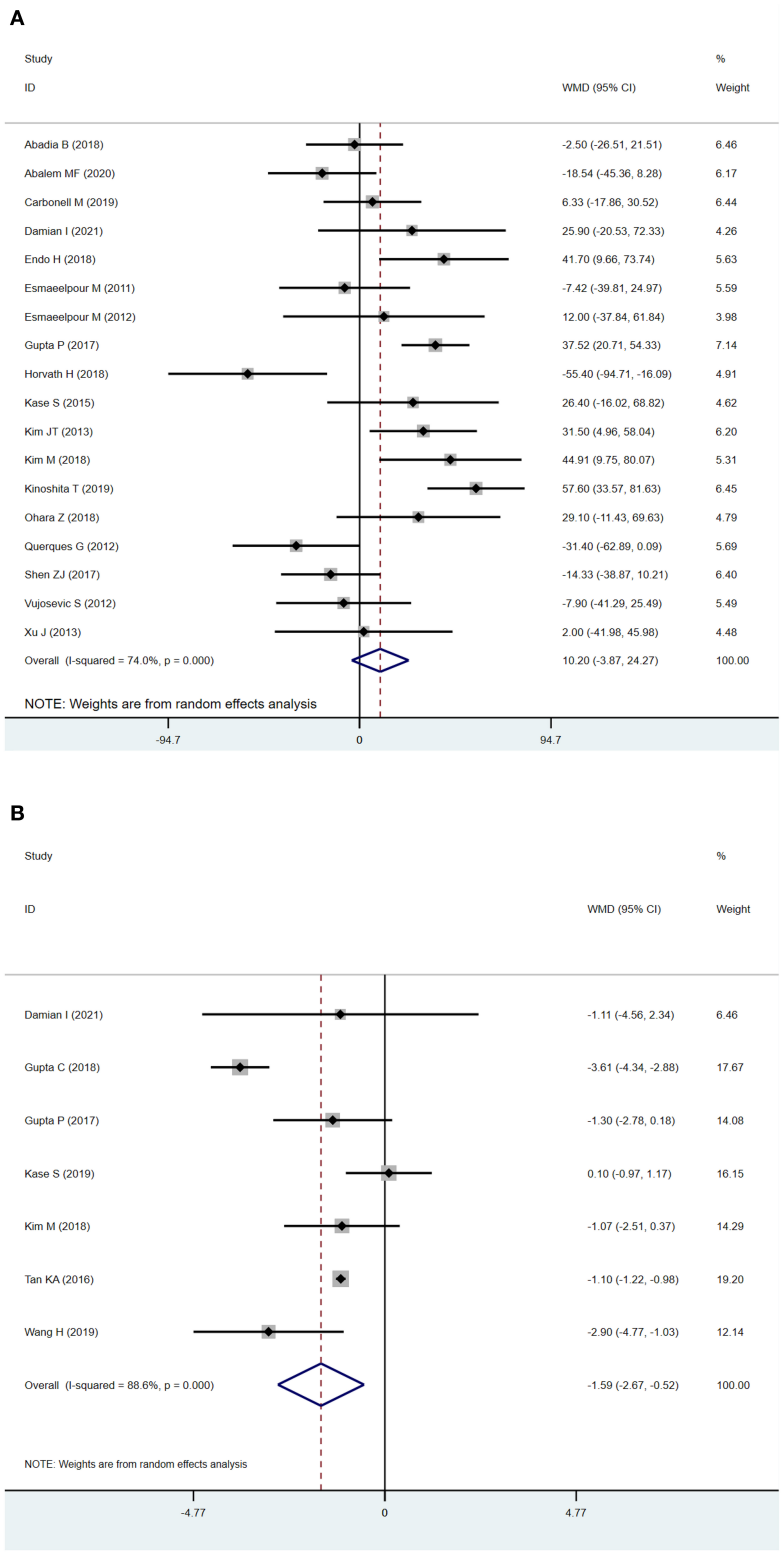
Subfoveal choroidal thickness (SFCT) and choroidal vascularity index (CVI) in patients with NDR and DR. **(A)** SFCT. **(B)** CVI.

The analysis of SFCT between patients with NDR and DR is described in Figure 2A. Eighteen studies were included in the evaluation of SFCT, and because of the substantial heterogeneity (*I*^2^ > 50%), random-effects models were used. The SFCT showed no significant difference between DR and NDR eyes (WMD = 10.20 μm; 95% CI:−3.87 to 24.27; *P* = 0.156, Figure 2A).

Seven studies used the CVI as the main outcome, and because the heterogeneity (*I*^2^ > 50%) was substantial, we used a random-effects model. The CVI was significantly lower in DR eyes compared to NDR eyes (WMD=-1.59; 95% CI:−2.67 to−0.52; *P* = 0.004, Figure 2B).

### Sub-analysis after adjusting for axial length

**Figure 3:**
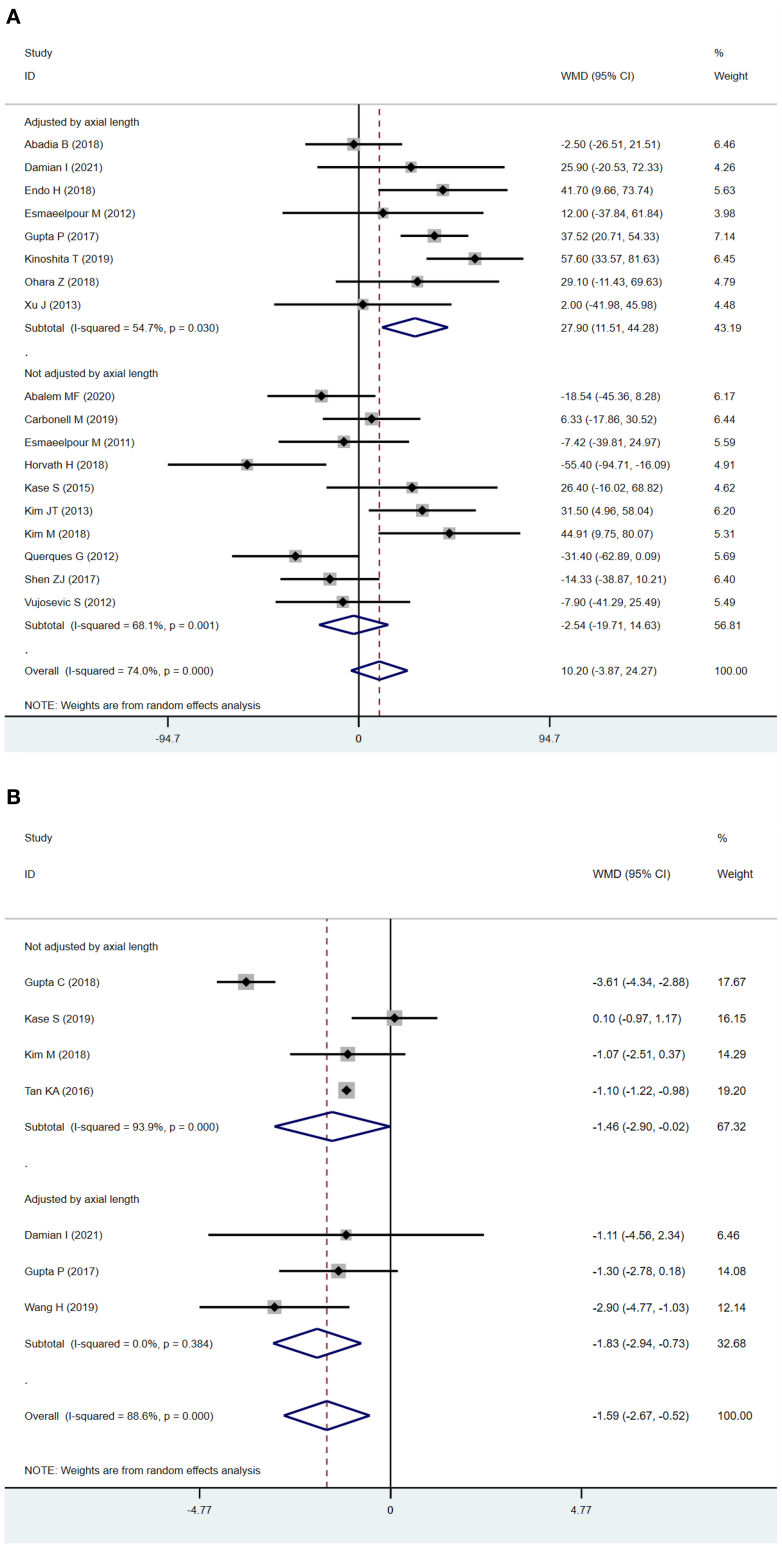
Sub-analysis of subfoveal choroidal thickness (SFCT) and choroidal vascularity index (CVI) according to the adjustment of axial length **(A)**. SFCT **(B)** CVI.

In subgroup analysis, we found SFCT was significantly increased in DR eyes after adjusting for axial length (adjusted by axial length: WMD = 27.90 μm; 95% CI: 11.51 to 44.28; *P* = 0.001, Figure 3A). The outcomes, with or without adjustment of axial length, diverged. Previous studies have shown that axial length can be an important confounding factor in measuring SFCT (31, 32), which might be one of the most serious limitations of the analysis. To ensure the quality of our meta-analysis, we excluded studies that measured SFCT without adjusting for axial length in this analysis. In analyzing of CVI, we found that the results after the adjustment of axial length were correlated with the main analysis (adjusted by axial length: WMD = −1.83, 95% CI = −2.94 to−0.73, *P* = 0.001, Figure 3B; main analysis: WMD = −1.59; 95%CI:−2.67 to−0.52; *P* = 0.004, Figure 2B), and the sub-analysis lowered the heterogeneity from 88.6 to 0.0%.

### Sub-analysis by OCT instrument

**Figure 4:**
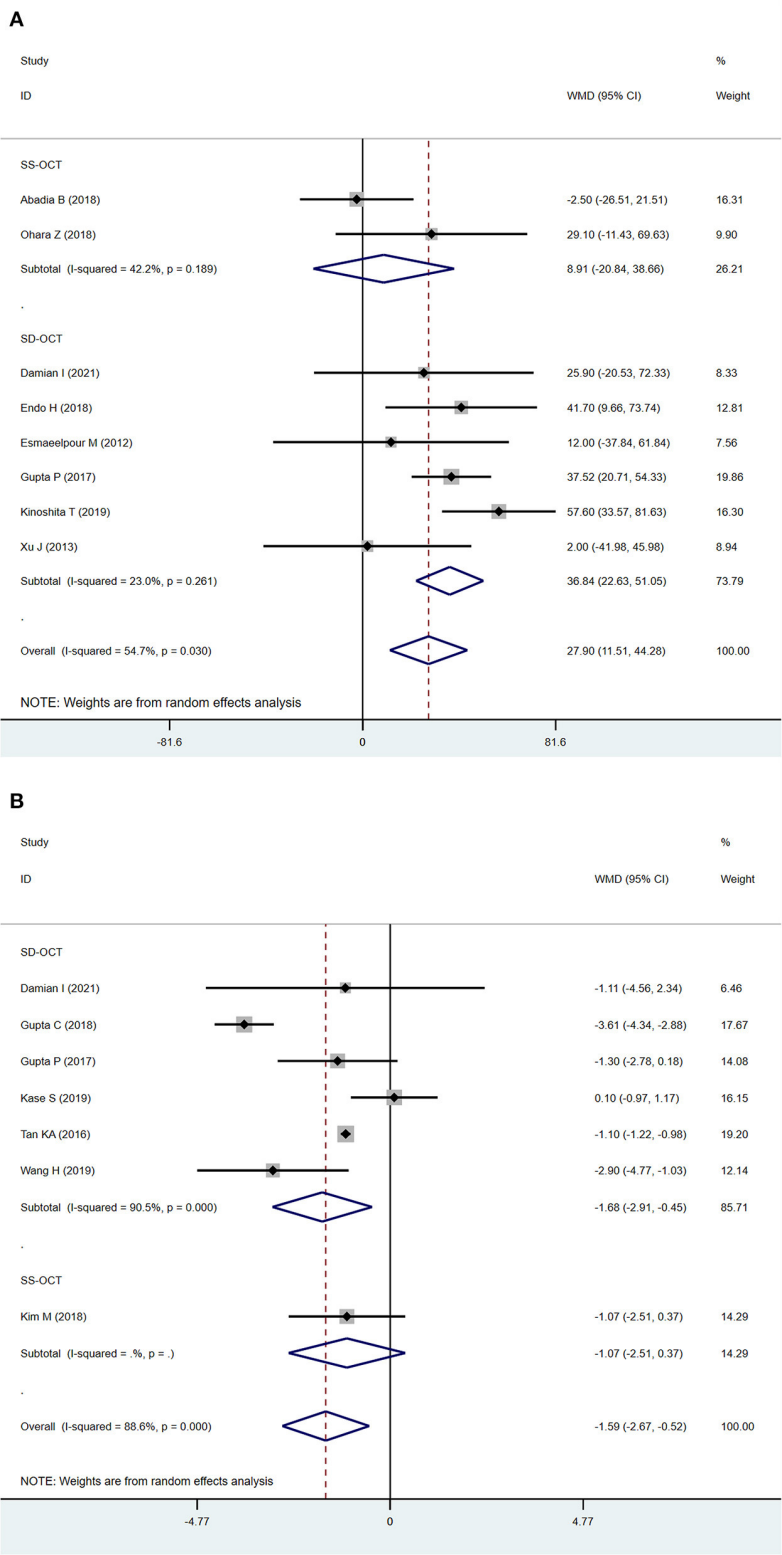
Sub-analysis of subfoveal choroidal thickness (SFCT) and choroidal vascularity index (CVI) according to OCT instrument **(A)** SFCT. **(B)** CVI.

A source of significant heterogeneity among studies may be due to the different OCT instruments used. The results from SD-OCT corresponded to the main analysis measuring SFCT and CVI (SFCT: WMD = 36.84 μm; 95% CI: 22.63–51.05; *P* < 0.001, Figure 4A; CVI: WMD = −1.68; 95%CI:−2.91 to−0.45; *P* = 0.007, Figure 4B). However, SS-OCT showed different results possibly due to the relatively small number of studies included (one study for CVI, two studies for SFCT).

### Comparison of DR stages to NDR

Because of disease progression and changes in fundus performance, DR can be divided into mild non-proliferative DR, moderate non-proliferative DR, severe non-proliferative DR (sNPDR), proliferative DR (PDR). Previous studies have shown that DR stage may have an influence on choroidal thickness (26, 28, 30) and CVI (5, 8, 23). To explore the choroidal structure changes in each DR stage, we pooled outcomes based on the different DR stages, including m-mNPDR (mild to moderate non-proliferative DR), sNPDR and PDR.

#### SFCT

**Figure 5:**
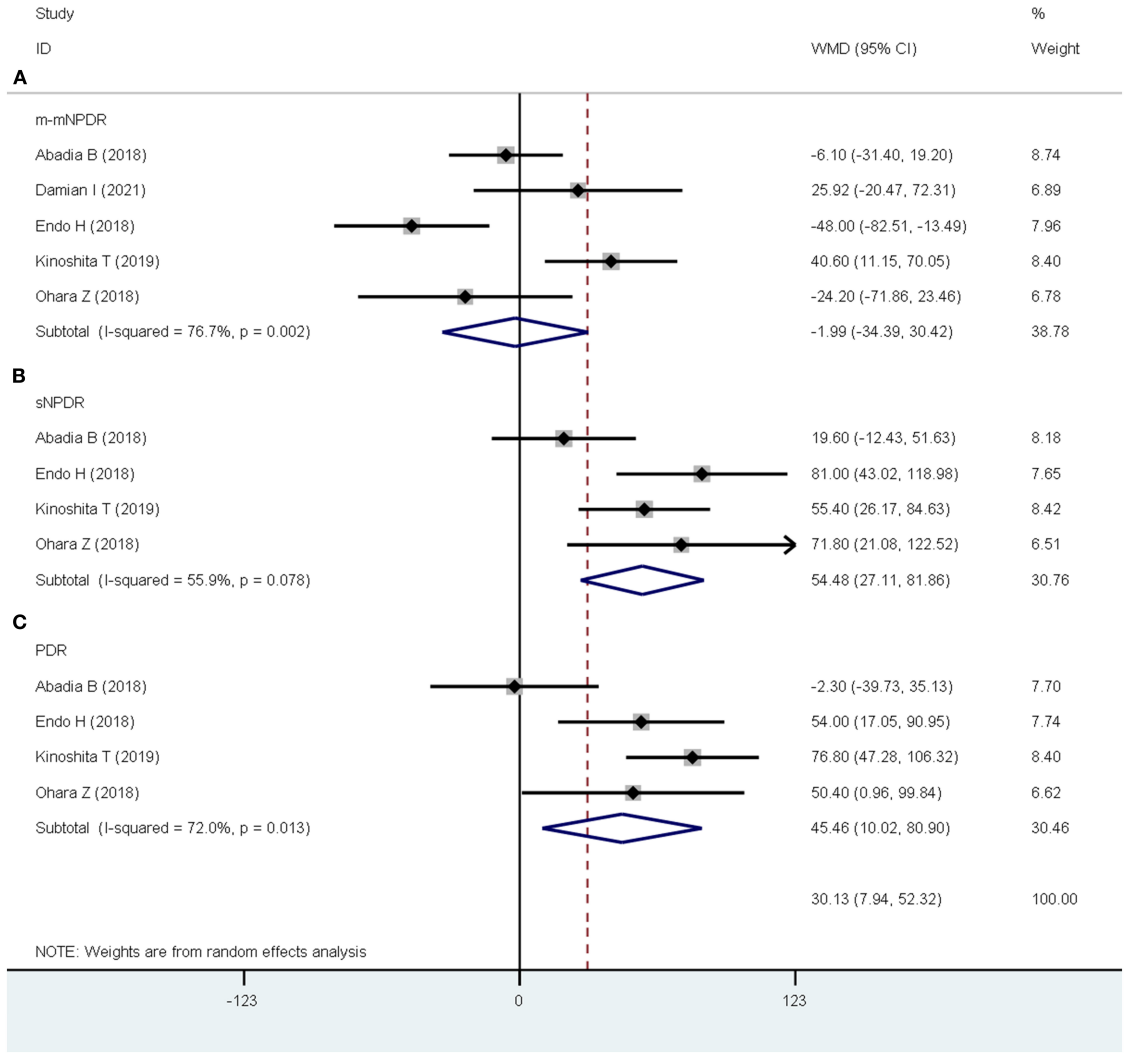
Subfoveal choroidal thickness (SFCT) in patients with NDR and different DR stages. **(A)** NDR and m-mNPDR. **(B)** NDR and sNPDR. **(C)** NDR and PDR.

The analyses revealed no significant difference in SFCT between NDR and m-mNPDR eyes (WMD = −1.99 μm; 95% CI:−34.39 to 30.42; *P* = 0.904, Figure 5A). The SFCT was significantly increased in sNPDR and PDR eyes (sNPDR: WMD = 54.48 μm; 95% CI: 27.11–81.86; *P* < 0.001, Figure 5B; WMD = 45.46 μm; 95% CI: 10.02–80.90; *P* = 0.012, Figure 5C).

#### CVI

**Figure 6:**
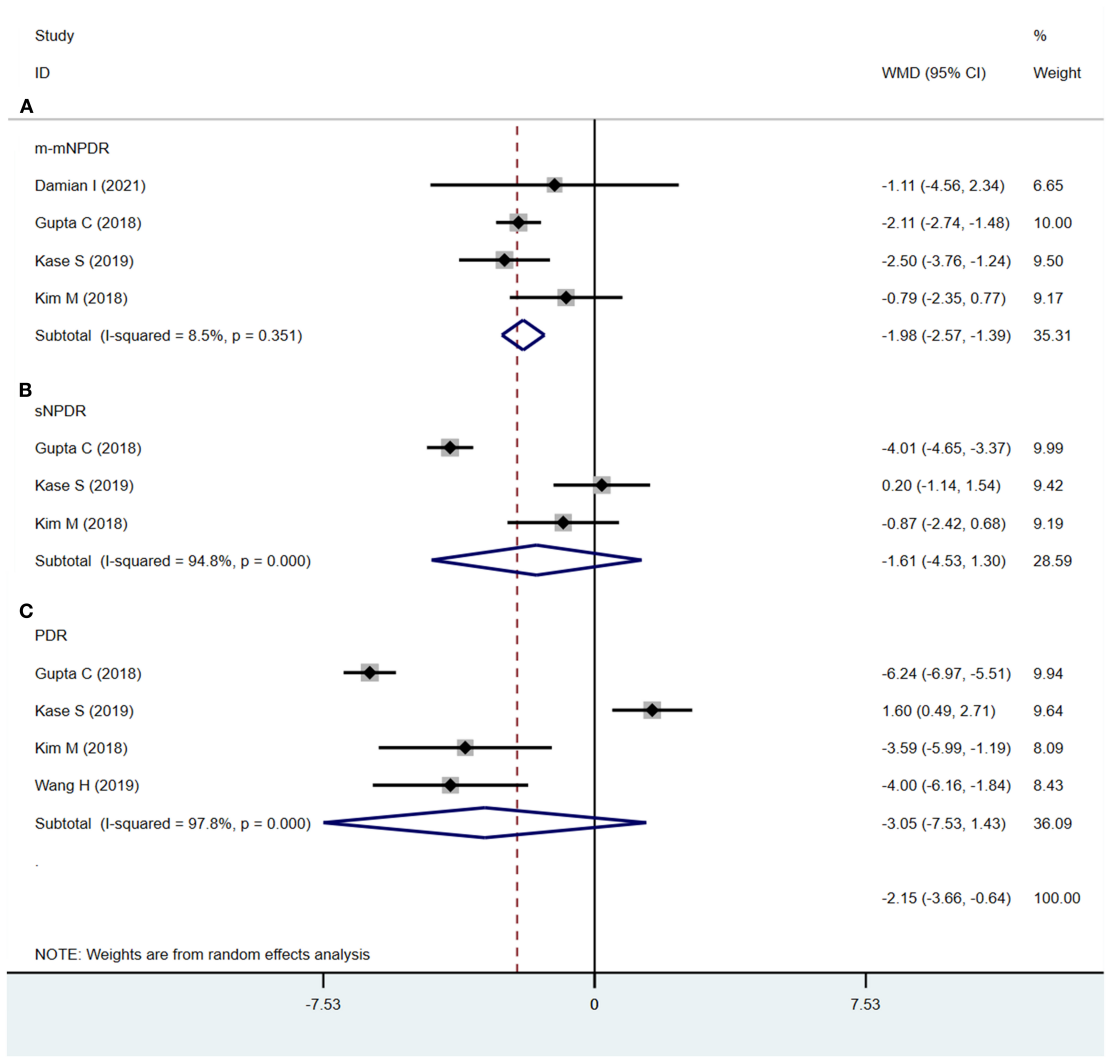
Choroidal vascularity index (CVI) in patients with NDR and different DR stages. **(A)** NDR and m-mNPDR. **(B)** NDR and sNPDR. **(C)** NDR and PDR.

The CVI was significantly decreased in m-mNPDR eyes compared to that in NDR eyes (WMD = −1.98; 95% CI:−2.57 to−1.39; *P* < 0.001, Figure 6A). Conversely, in sNPDR and PDR, there were no significant differences compared that in to NDR (sNPDR: WMD = −1.61; 95%CI:−4.53 to 1.30; *P* = 0.279, Figure 6B; PDR: WMD = −3.05; 95% CI:−7.53 to 1.43; *P* = 0.183, Figure 6C).

### Sensitivity analysis

“Leave-one-out” sensitivity analyses were generated to evaluate the influence of a single study on the pooled results (Supplementary Figures S1–S3), and there were no obvious changes in the results when any particular study was removed.

The “leave-one-out” sensitivity analysis revealed that the study by Abadia et al. (15) contributed mostly to the heterogeneity in SFCT (Table 3). After excluding the study low heterogeneity was noted (total DR, 10%; Supplementary Figure S7A; sNPDR, 0%; Supplementary Figure S7B; PDR, 0%; Supplementary Figure S7C). In the sensitivity analysis of CVI (Table 4), excluding the study by Gupta et al. (23) significantly lower the heterogeneity (total DR, 41%; Supplementary Figure S8A; sNPDR, 4.7%; Supplementary Figure S8B).

**Table 3:** Sensitivity analysis of SFCT in DR patients.

**Study**	**Random-effects model**		**Heterogeneity**
	**WMD**	**(95% CI)**	***I^2^***
**1.NDR and DR**			
Abadia et al. (15)	**0.49693599**	**[0.33351207** **0.66035992]**	**10%**
Damian et al. (21)	0.40553012	[0.2521773 0.55888301]	60%
Endo et al. (11)	0.38117513	[0.22636071 0.53598958]	60%
Esmaeelpour et al. (16)	0.40702617	[0.25643048 0.5576219]	61%
Gupta et al. (24)	0.3118968	[0.14181076 0.48198283]	56%
Kinoshita et al. (27)	0.31105003	[0.15072405 0.47137603]	58%
Ohara et al. (28)	0.39742377	[0.24575442 0.54909313]	37%
Xu et al. (14)	0.44536459	[0.28878689 0.6019423]	61%
**2. NDR and m-mNPDR**			
Abadia et al. (15)	−1.0095644	[-46.60305 44.58392]	82%
Endo et al. (11)	10.313095	[-18.883085 39.509277]	63%
Ohara et al. (28)	2.607975	[-35.286579 40.502529]	81%
Damian et al. (21)	−7.9985437	[-45.738407 29.74132]	81%
Kinoshita et al. (27)	−14.417554	[-42.992428 14.15732]	58%
**3. NDR and sNPDR**			
Abadia et al. (15)	**66.109703**	**[45.037811** **87.181595]**	**0%**
Endo (11)	45.683723	[16.45019 74.917252]	50%
Kinoshita et al. (27)	55.419155	[13.475044 97.363266]	67%
Ohara et al. (28)	50.947495	[17.634071 84.260918]	70%
**4. NDR and PDR**			
Abadia et al. (15)	**64.78746**	**[43.885159** **85.689766]**	**0%**
Endo (11)	42.168507	[-8.4728012 92.809814]	81%
Ohara et al. (28)	43.815971	[-2.2589738 89.890915]	81%
Kinoshita et al. (27)	32.887196	[-4.8808665 70.655258]	61%

SFCT, subfoveal choroidal thickness; NDR, diabetic eyes with no retinopathy; DR, diabetic retinopathy; m-mNPDR, mild to moderate non-proliferative diabetic retinopathy; sNPDR, severe non-proliferative diabetic retinopathy; PDR, proliferative diabetic retinopathy.

The bold values indicate the studies contributed most to heterogeneity.

**Table 4:** Sensitivity analysis of CVI in DR patients.

**Study**	**Random-effects model**		**Heterogeneity**
	**WMD**	**(95% CI)**	***I^2^***
**1. NDR and DR**			
Damian et al. (21)	−1.626483	[-2.7562518 −0.49671403]	91%
Gupta et al. (23)	**-1.0485296**	**[-1.6540127** **−0.44304654]**	**41%**
Gupta et al. (24)	−1.6400692	[-2.8651676 −0.4149709]	91%
Kase et al. (25)	−1.9153022	[-3.1549792 −0.67562515]	89%
Kim et al. (12)	−1.6794566	[-2.9098058 −0.44910744]	91%
Tan et al. (8)	−1.6903533	[-3.2109909 −0.16971554]	86%
Wang et al. (5)	−1.4112904	[-2.5666049 −0.25597602]	90%
**2.NDR and m-mNPDR**			
Kase et al. (25)	−1.7349782	[-2.5982447 −0.87171167]	22%
Damian et al. (21)	−1.9658893	[-2.719326 −1.2124527]	34%
Kim et al. (12)	−2.159235	[-2.7122207 −1.6062492]	0%
Gupta et al. (23)	−1.6746107	[-2.9174035 −0.43181807]	32%
**3. NDR and sNPDR**			
Kase (25)	−2.0167465	[-4.4330173 0.39952454]	91%
Kim (12)	−1.6256723	[-4.56036 1.3090153]	95%
Gupta et al. (23)	**-0.44054857**	**[-1.3013378** **0.4202407]**	**4.7%**
**4. NDR and PDR**			
Kase (25)	−4.869297	[-6.7858934 −2.9527009]	73%
Wang et al. (5)	−2.7391636	[-8.4354811 2.9571536]	99%
Kim (12)	−2.875267	[-8.4481916 2.6976573]	99%
Gupta et al. (23)	−1.9022373	[-6.0220013 2.2175269]	93%

CVI, choroidal vascularity index; NDR, diabetic eyes with no retinopathy; DR, diabetic retinopathy; m-mNPDR, mild to moderate non-proliferative diabetic retinopathy; sNPDR, severe non-proliferative diabetic retinopathy; PDR, proliferative diabetic retinopathy.

The bold values indicate the studies contributed most to heterogeneity.

### Publication bias

Potential publication bias was evaluated using funnel plots (Supplementary Figures S4–S6). No obvious asymmetry was observed. Publication bias was also calculated using Begg's and Egger's tests (Table 5), and no obvious evidence of publication bias was found.

**Table 5:** Begg's and Egger's tests results for the evaluation of publication bias.

**Outcome indicators**	**No**.	**Begg's test**		**Egger's test**	
		**z**	**Pr > |z|**	***t***	**P > |t|**
**1.SFCT**
NDR vs. DR	8	0.75	0.452	−0.75	0.494
NDR vs. m-mNPDR	5	0.24	0.806	−0.23	0.832
NDR vs. sNPDR	4	0.34	0.734	0.80	0.510
NDR vs. PDR	4	1.02	0.308	−0.66	0.579
**2.CVI**
NDR vs. DR	7	0.90	0.386	−0.78	0.443
NDR vs. m-mNPDR	4	0.34	0.734	0.87	0.477
NDR vs. sNPDR	3	0.00	1.000	3.05	0.202
NDR vs. PDR	4	0.34	0.734	0.51	0.663

SFCT, subfoveal choroidal thickness; NDR, diabetic eyes with no retinopathy; DR, diabetic retinopathy; m-mNPDR, mild to moderate non-proliferative diabetic retinopathy; sNPDR, severe non-proliferative diabetic retinopathy; PDR, proliferative diabetic retinopathy; CVI, choroidal vascularity index.

## Discussion

In the present study, we pooled the SFCT and CVI of DR patients and compared them with those of NDR patients. We also pooled SFCT and CVI for m-mNPDR, sNPDR, and PDR, respectively. Resultantly, we found that the CVI was decreased in DR patients, and the SFCT was thicker in DR patients after adjusting for axial length. Moreover, we found that the CVI was significantly decreased in m-mNPDR, and SFCT was significantly thicker in sNPDR and PDR.

Despite technological advances, the pathophysiology of DR remains unclear. Dysfunction of the inner blood-retinal barrier is an early event in the development of DR (33). However, diabetic choroidopathy also plays an important role in the pathogenesis of DR because the choroid provides oxygen and nutrients to the outer retinal layers and is the only blood supply for the avascular fovea (20). Animal model suggested reduced choroidal blood could be an early pathological change in diabetic retinopathy (34). Histological analyses have shown that choriocapillaris degeneration and extensive dropout of the choriocapillaris are present in the initial stage of DR (35–37). Other vascular changes (13, 38) such as microaneurysms, non-perfusion areas, narrowing of vascular lumens, and neovascularization were also found in the choroid of diabetic eyes. The level of leukocyte adhesion molecules is elevated in the choroidal blood vessels of DM patients (39). In addition to histological analyses, changes in choroidal hemodynamics can occur even before the onset of DR in DM patients. Nagaoka et al. (40) showed a decreased choroidal blood flow in the foveal region in patients with diabetes. Choroidal parameters SFCT and CVI included in our study help to provide a better understanding of the pathophysiology of DR.

The choroid is the only blood supply to the avascular fovea (20) and the distribution profiles of choroidal thickness in persons are similar: thickness subfoveally and thinnest nasally. This makes the choroidal thickness of the subfoveal region more sensitive to minor pathological changes than other regions. However, our results failed to show any difference of SFCT between NDR and DR initially. Previous studies have already described that choroidal thickness is related to several factors, such as age (41), sex (31), DR stage (10, 13, 20, 28), OCT instruments and axial length may also be potential confounders. However, CVI may be a more stable parameter in indicate choroidal structure which is less likely to be impacted by these potential confounders (42). To explore the influence of the confounding factors, we conducted sub-group analyses based on the axial length and OCT instrument.

The studies included in this meta-analysis defined CVI as the ratio of the luminal area (LA) to the total choroidal area in the subfoveal choroidal area with a width of 1,000–1,500 μm, centered at the fovea. The CVI can indirectly quantitively measure choroidal vascularity, overcoming the limitations of using choroidal thickness alone (12).

In the subgroup analysis adjusted for axial length, we demonstrated that SFCT was significantly increased in DR eyes which was different from the main analysis indicating a potential influence of axial length on SFCT. Previous studies fully described the importance of axial length in estimating choroidal thickness and found a negative correlation between foveal choroidal thickness and axial length (43–45). To ensure the quality of our meta-analysis, we then excluded studies that measured SFCT without adjusting for axial length. The sub-analysis of CVI results showed a similar trend to the main analysis, which did not support the axial length to be a potential confounder in measuring CVI.

The diameter of choroidal vessels reduces in diabetes mellitus patients before retinopathy manifestation due to vascular constriction secondary to choroidal hypoxia, and changes of choroidal blood flow (35, 40). There are numbers of studies showed a significant decrease in SFCT and CVI in the diabetes mellitus patients compared to healthy controls (5, 6, 8, 12, 15, 24, 29, 42, 46, 47). Our results showed that CVI was significantly decreased in m-mNPDR compared with NDR, indicating that the ischemic change and reduce of blood flow in the choroidal vasculature continues to progress in early stage of DR. The degeneration of choriocapillaries and reduce of blood flow in the diabetic choroid could induce hypoxia in the RPE (48), and upregulate vascular endothelial growth factor (VEGF) production caused by RPE hypoxia, thus stimulating angiogenesis (49). We hypothesized that choroidal neovascularization caused by elevated VEGF may explain the increase in SFCT in later stage of DR. Elevated VEGF causes choroidal vasodilation, elevation of the choroidal blood flow, and vascular permeability which contributes to both stromal and luminal components of choroid and subsequently increases the choroidal thickness (24). And anti-VEGF therapy significantly decrease the choroidal thickness in DR patients (50). Choroidal neovascularization contributes to the stromal component while choroidal vasodilation increases the luminal component. Due to the decrease in CVI, we consider the increase is mainly in the stromal component. In short, choroidal vascular constriction and low blood flow reduce the luminal component of the choroid and thereby decreasing CVI, whereas proliferative changes in later DR stages increase the stromal component and resulting in an increased SFCT.

One of the main challenges of the study was controlling the heterogeneity of the existing studies and confounders. To our knowledge, confounders for measuring choroidal thickness by OCT include age (41), gender, axial length (31), circadian rhythm (51), OCT instrument.

The development of SS-OCT and SD-OCT has greatly facilitated choroid imaging. EDI-OCT improves the visualization of the choroidoscleral junction, and the choroidal thickness can now be visualized and measured (52). In contrast to the usual wavelengths for SD-OCT, SS-OCT uses a longer wavelength to achieve greater tissue penetration, and the swept source approach features less signal roll off (53), thus allowing better visualization of both retinal and choroidal anatomy than SD-OCT. To explore the heterogeneity across different OCT instruments for the measurement of CVI and SFCT in DR, we conducted a subgroup analysis according to OCT type. The SFCT and CVI derived from SD-OCT corresponded to the main analysis. However, the SFCT and CVI with SS-OCT showed a different result, possibly due to the relatively small number of included studies (one study for CVI, two for SFCT).

Although our meta-analysis has important strengths, its potential limitations should be noted. First, significant heterogeneity was observed in the primary analysis. To elucidate the source of the heterogeneity, we performed sensitive analysis. Studies by Abadia et al. (15) and Gupta et al. (23) may contribute mostly to the heterogeneity in SFCT and CVI, respectively. Second, we only included published data; thus, a potential publication bias may remain, although there is no clear evidence of publication bias as revealed by our Begg's and Egger's test results. Third, patients with later-stage DR typically prefer to undergo systematic treatment such as glycemic control (2, 26, 54) and are more likely to receive focal treatment such as intraocular injection (50) and pan retinal photocoagulation (PRP) (55–57), which may potentially influence the SFCT and CVI. Previous studies showed that PRP may alter the SFCT at different times after treatment. The SFCT increases 1 week after PRP (55), since damage to the peripheral choriocapillaris causes peripheral choroidal blood flow to decrease and redistributes the blood flowing from the peripheral to foveal Centralia (58). However, SFCT also decreases at one (55, 56) and 3 months (55, 59) after PRP because of the reduction in total choroidal capillary blood flow. Although, we have excluded patients who received focal treatment for DR in the last 3 months, a potential bias may remain especially in sNPDR and PDR. Fourth, DM type, disease duration, and HbA1c level (60) can affect the SFCT. Permanent high blood glucose level in diabetic patients with inadequate treatment may lead to choroidal vascular damage and cause choroidal thinning (26). Poorly controlled HbA1c is also associated with more severe stages of DR (61); this may be due to the lower choroidal blood flow and choroidal hypoxia in the higher concentrations of blood sugar (62). However, we were unable to conduct these subgroup analyses because of lack of corresponding data.

## Conclusion

We described the changes in the SFCT and CVI in DR. CVI and SFCT displayed a significant decrease and increase respectively, indicating low perfusion and proliferative changes are the major pathological changes in choroid. Moreover, CVI and SFCT might be valuable parameters for discriminating DR from NDR. This facilitates a better understanding of the role of the choroid in the pathophysiology of DR and provides references for ophthalmologists to detect the onset of DR in DM patients.

## Data availability statement

The original contributions presented in the study are included in the article/Supplementary material, further inquiries can be directed to the corresponding author.

## Author contributions

BJ: study design and concept. JJ, JL, and JY: database search and data extracting. JJ: data analysis and manuscript writing. All authors contributed to the article and approved the submitted version.

## Funding

This study was supported by the Natural Science Foundation of China (NSFC 82070967 and 81770930 to BJ). The sponsors did not participate in the design or implementation of this study.

## Conflict of interest

The authors declare that the research was conducted in the absence of any commercial or financial relationships that could be construed as a potential conflict of interest.

## Publisher's note

All claims expressed in this article are solely those of the authors and do not necessarily represent those of their affiliated organizations, or those of the publisher, the editors and the reviewers. Any product that may be evaluated in this article, or claim that may be made by its manufacturer, is not guaranteed or endorsed by the publisher.
