# Supplementary material for: Optical coherence tomography evaluation of choroidal structure changes in diabetic retinopathy patients: A systematic review and meta-analysis
Source: Front Med (Lausanne). 2022 Oct 20;9:986209. doi: 10.3389/fmed.2022.986209 (PMC9630657; doi:10.3389/fmed.2022.986209)
Supplement: Supplementary file 1 [file Data_Sheet_1.PDF]

Supporting information

Figure legends:

Figure S1. Sensitivity analysis of subfoveal choroidal thickness (SFCT) in patients with NDR and DR.

Figure S2. Sensitivity analysis of subfoveal choroidal thickness (SFCT) **after adjusting for axial length** in patients with NDR and different DR stages. **(A)** NDR and DR. **(B)** NDR and m-mNPDR. **(C)** NDR and sNPDR. **(D)** NDR and PDR.

Figure S3. Sensitivity analysis of choroidal vascularity index (CVI) in patients with NDR and different DR stages. **(A)** NDR and DR. **(B)** NDR and m-mNPDR. **(C)** NDR and sNPDR. **(D)** NDR and PDR.

Figure S4. Funnel plot of subfoveal choroidal thickness (SFCT) in patients with NDR and DR.

Figure S5. Funnel plot of subfoveal choroidal thickness (SFCT) after adjusting for axial length in NDR and different DR stages. **(A)** NDR and DR. **(B)** NDR and m-mNPDR. **(C)** NDR and sNPDR. **(D)** NDR and PDR.

Figure S6. Funnel plot of choroidal vascularity index (CVI) in patients with NDR and different DR stages. **(A)** NDR and DR. **(B)** NDR and m-mNPDR. **(C)** NDR and sNPDR. **(D)** NDR and PDR.

Figure S7. SFCT in patients with NDR and DR after excluding the study of **Abadia et al**. **(A)** NDR and DR. **(B)** NDR and sNPDR. **(C)** NDR and PDR

Figure S8. CVI in patients with NDR and DR after excluding the study of **Gupta et al**. **(A)** NDR and DR. **(B)** NDR and sNPDR.

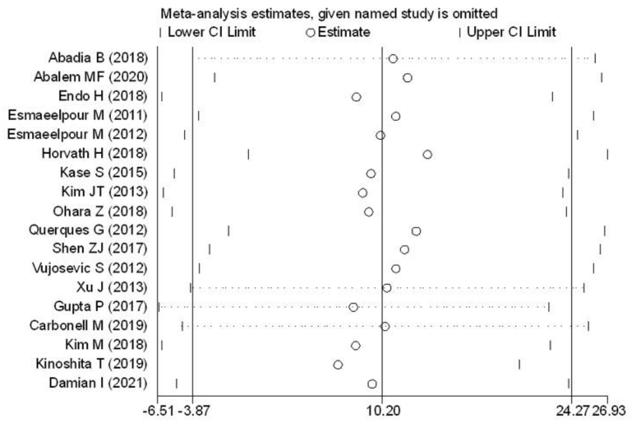

Figure S1. Sensitivity analysis of subfoveal choroidal thickness (SFCT) in patients with NDR and DR.

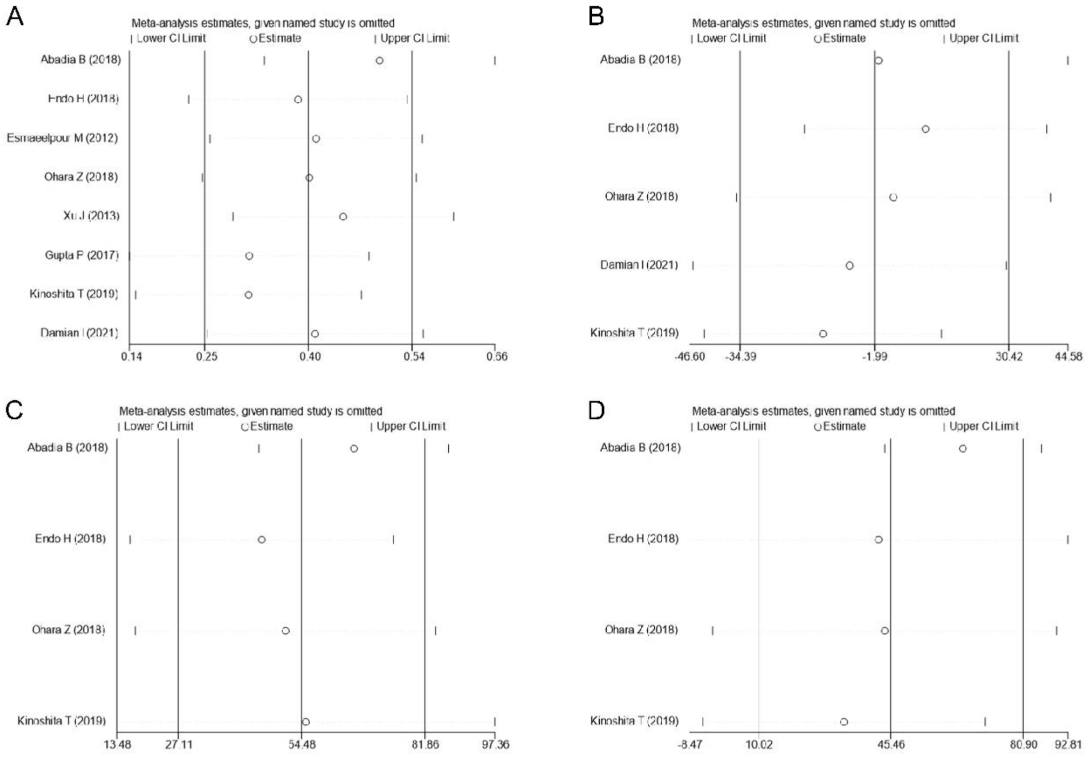

Figure S2. Sensitivity analysis of subfoveal choroidal thickness (SFCT) **after adjusting for axial length** in patients with NDR and different DR stages. **(A)** NDR and DR. **(B)** NDR and m-mNPDR. **(C)** NDR and sNPDR. **(D)** NDR and PDR.

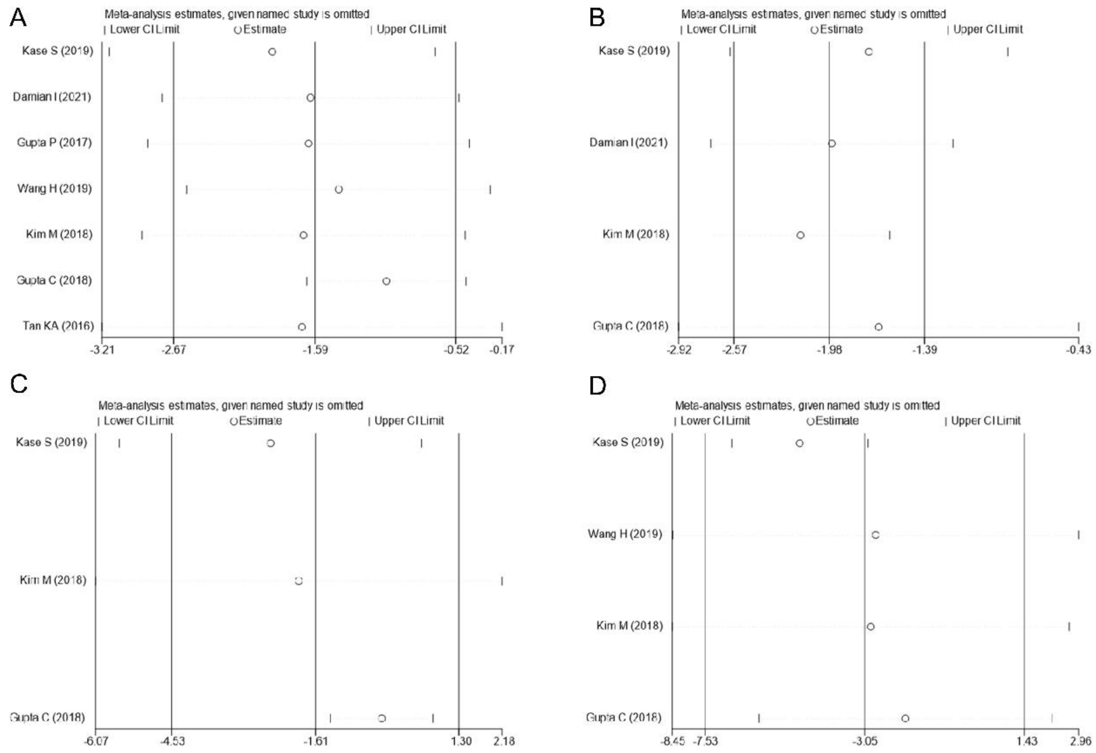

Figure S3. Sensitivity analysis of choroidal vascularity index (CVI) in patients with NDR and different DR stages. **(A)** NDR and DR. **(B)** NDR and m-mNPDR. **(C)** NDR and sNPDR. **(D)** NDR and PDR.

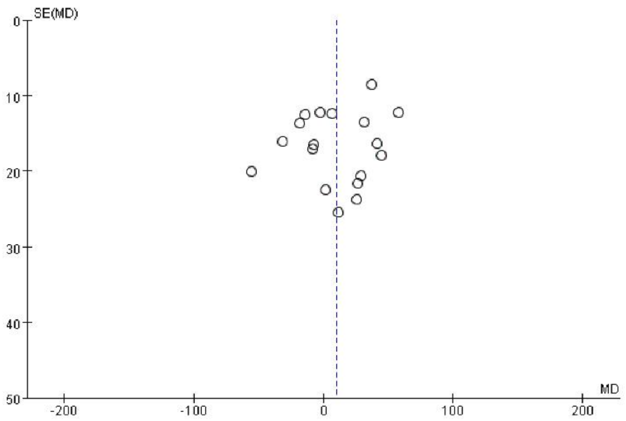

Figure S4. Funnel plot of subfoveal choroidal thickness (SFCT) in patients with NDR and DR.

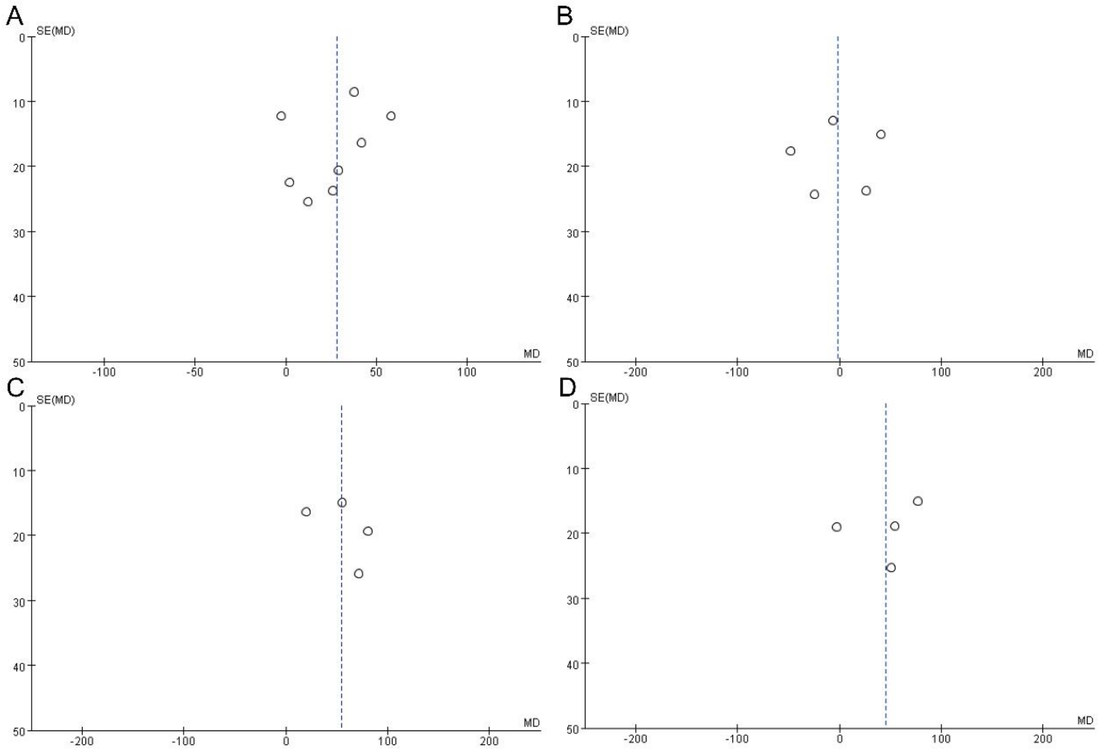

Figure S5. Funnel plot of subfoveal choroidal thickness (SFCT) **after adjusting for axial length** in NDR and different DR stages. **(A)** NDR and DR. **(B)** NDR and m-mNPDR. **(C)** NDR and sNPDR. **(D)** NDR and PDR.

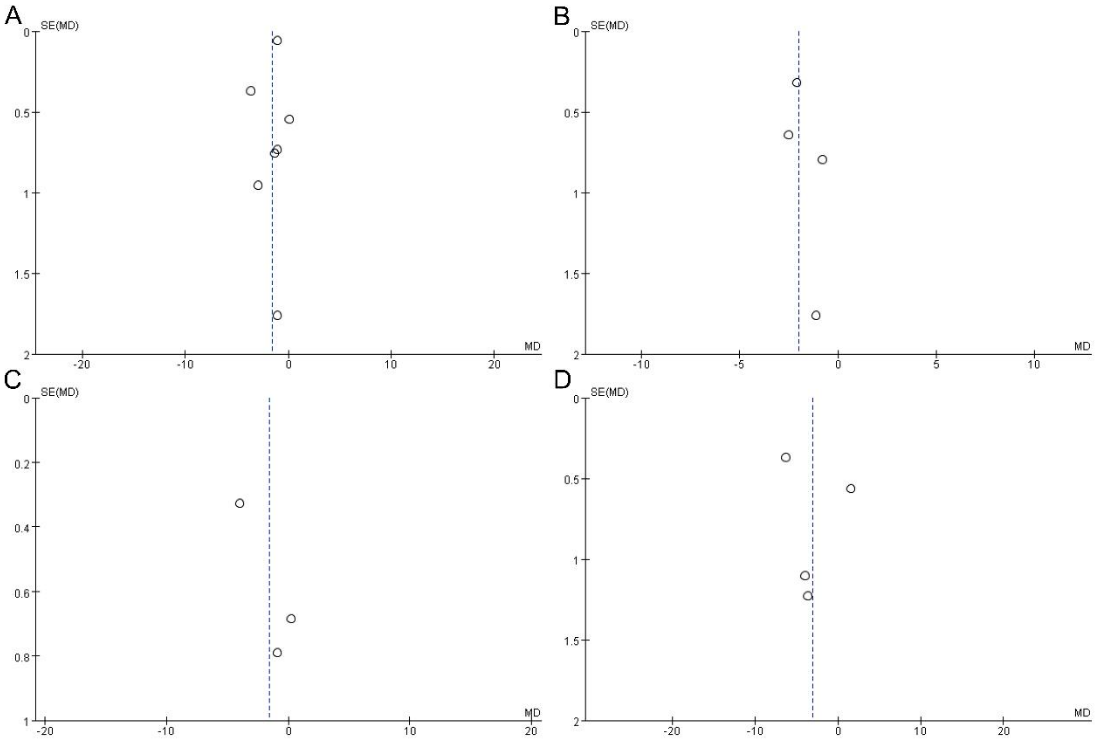

Figure S6. Funnel plot of choroidal vascularity index (CVI) in patients with NDR and different DR stages. **(A)** NDR and DR. **(B)** NDR and m-mNPDR. **(C)** NDR and sNPDR. **(D)** NDR and PDR.

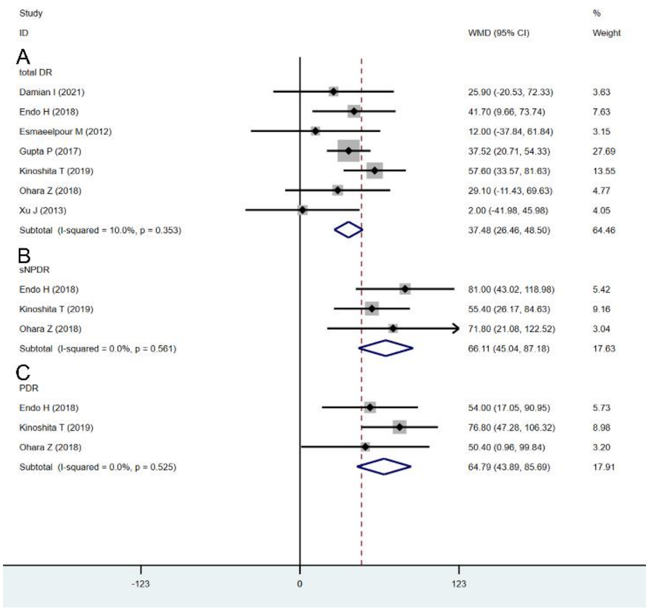

Figure S7. SFCT in patients with NDR and DR after excluding the study of **Abadia et al**. **(A)** NDR and DR. **(B)** NDR and sNPDR. **(C)** NDR and PDR

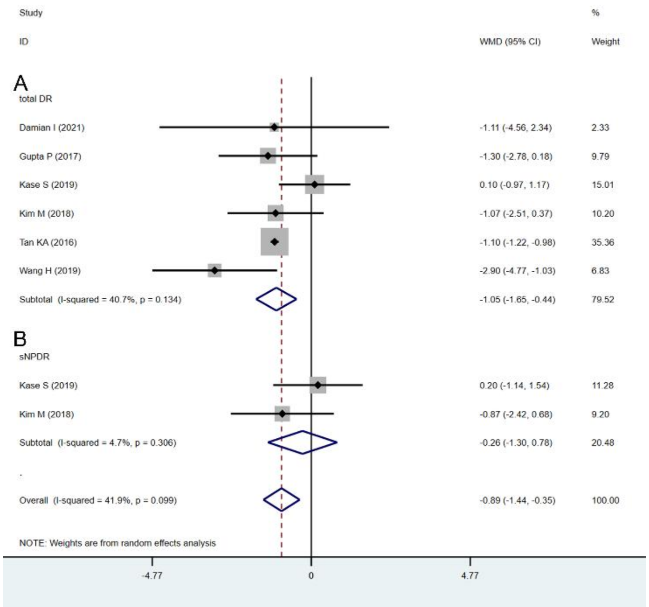

Figure S8. CVI in patients with NDR and DR after excluding the study of **Gupta et al**. **(A)** NDR and DR. **(B)** NDR and sNPDR.
